# FIRE Stones: impact of forced diuresis on the residual fragment rate after flexible ureteroscopy for destruction of kidney stones with laser—protocol for a randomized controlled two-parallel group multicenter trial with blinding evaluation

**DOI:** 10.1186/s13063-024-08309-0

**Published:** 2024-07-04

**Authors:** ML. Letouche, B. Giraudeau, MS. Agier, F. Bruyere

**Affiliations:** 1 Department of Urology, CHRU Tours, 37000 Tours, France; 2 Clinical Investigation Center, INSERM 1415, CHRU Tours, 37000 Tours, France; 3 Pharmacovigilance Regional Centre (CRPV), CHRU Tours, 37000 Tours, France

**Keywords:** Kidney stones, Flexible ureteroscopy, Forced diuresis, Furosemide, Stone-free rate, Residual fragments, Lithiasis patient, Randomized trial

## Abstract

**Background:**

Lithiasis is a common and recurrent disease. Flexible ureteroscopy (fURS) is the cornerstone of laser treatment of kidney stones. Kidney stones destruction requires its laser pulverization into small fragments in order to remove them through the ureter or improve their spontaneous expulsion along the urinary tract. However, most of the time, all the micro-fragments and dust created cannot be extracted using our surgical tools and may stay intra-renally at the end of the procedure. Adjuvant treatments (such as forced diuresis, inversion or mechanical pressure) were previously described to improve the expulsion of stone fragments after extra-corporeal shock wave lithotripsy. Nevertheless, the impact of adjuvant treatment after fURS remains unclear and mainly theoretical.

**Objective:**

The primary objective is to show that the injection of 40 mg of furosemide in slow intravenous during 10 min, after the procedure, increases the stone-free rate 3 months after a fURS for destruction of kidney stones with laser.

**Methods/design:**

The study will be a two-parallel group randomized, controlled, multicentric trial with a blinding evaluation. Nine French departments of urology will participate. Patients will be randomized in 2 groups: the experimental group (injection of 40 mg of furosemide at the end of the surgery) and a control one (usual care). Patients will be followed up for 3 months (± 2 weeks) after the surgery. Then, we will perform a low dose abdomino-pelvic CT scan. The primary outcome is the stone-free rate at 3 months. A centralized review of the images will be performed by two specialized radiologists, in a blind and crossed way to allow a homogenization of the results. The secondary outcomes will include the rate of early post-operative urinary tract infection (UTI), the evaluation of post-operative pain, and the safety of the use of furosemide in patients treated by fURS for renal stone laser destruction. As secondary objectives, it is also planned to look at the effect of the prescription of an alpha-blocker as usual treatment on stone-free rate and to assess the agreement between the imaging analysis of the urologist and the specialized radiologist.

**Discussion:**

Lithiasis is a public health problem. It affects about 10% of the general population. This prevalence is increasing (multiplied by 3 in 40 years), partly due to changes in the population’s eating habits over the years. The lithiasis patient is a patient with a chronic disease requiring annual follow-up and who may suffer from multiple recurrences, with a recurrence rate at 5 years of 50%.

Recurrences are partly due to residual fragments left in the kidneys at the end of the operation. Other risk factors for recurrence include dietary hygiene and the presence of an associated metabolic disease. The metabolic blood and urine tests recommended by the Association Française d’Urologie (AFU) can be used to manage these last two problems.

As far as residual fragments are concerned, their presence leads to an early recurrence of stones because they form the bed for a new aggregation of crystals in the kidneys. Being able to reduce the rate of residual fragments in patients with the use of furosemide at the end of the intervention therefore seems essential in the management of recurrences in our patients. This will also improve our patients’ quality of life.

Indeed, lithiasis disease leads to chronic pain associated with acute pain that motivates consultations to the emergency for specialized management. This study is the first to evaluate the impact of forced diuresis with the use of furosemide on the stone-free rate after a fURS for destruction of kidney stone with laser.

**Trial registration:**

ClinicalTrials.gov Identifier: NCT05916963, first received: 22 June 2023.

EU Clinical Trials Register EudraCT Number: 2022-502890-40-00.

**Supplementary Information:**

The online version contains supplementary material available at 10.1186/s13063-024-08309-0.

## Background

### Background and rational

Lithiasis is a public health problem. It affects about 10% of the general population. This prevalence is increasing (multiplied by 3 in 40 years), partly due to changes in the population’s eating habits over the years [1]. The risk factors for the development of urinary stones are multiple: socio-economic status, environmental factors, genetic predisposition, and certain metabolic disorders [2]. The increase in metabolic syndrome plays a large role in the development of lithiasis disease in developed countries. The management of lithiasis is extensive. It ranges from active surveillance with or without expulsive treatment (alpha-blocker) to surgical removal of stones (flexible ureteroscopy [fURS] and percutaneous nephrolithotomy [PCNL]) and also includes extra-corporeal shock wave lithotripsy (SWL) [2]. The size, location, and density of the stone influence the choice of treatment [3, 4].

Over the past 20 years, fURS have dramatically changed the management of kidney stones and has become the most performed procedure for the removal and destruction of kidney stones. More than 40,000 fURS for stones are performed per year in France in common practice.

The lithiasis patient is a patient with a chronic disease requiring annual follow-up and who may suffer from multiple recurrences, with a recurrence rate at 5 years of 50% [1, 2].

Being able to reduce the rate of residual fragments in patients might reduce the risk of recurrence and therefore improve the quality of life. All this leads to the need to evaluate the interest of an adjuvant treatment associated with fURS (being the predominant treatment for kidney stones) to further decrease the stone-free rate in these lithiasis patients.

In the literature, we find as adjuvant treatments: post-urotherapy and forced diuresis.

The goal of post-urotherapy is to relocate stones trapped in the lower or middle calyces into the renal pelvis in order to increase their spontaneous clearance by the natural route. This can be done for small stones immediately or after SWL, fURS, or PCNL for residual fragments. The patient is placed in a special position (lateral safety position with Trendelenburg in the prone position at − 30°) allowing the orientation of the caliceal stem of the inferior and anterior calyx to be favorable to the evacuation of the stones. Mechanical percussion-vibration is then performed. A preliminary per os hydration is associated to increase the diuresis.

A retrospective, single-center study conducted by Lechevallier et al. in Marseille in 2015 [5] showed that it is a non-invasive, non-morbid, and well-tolerated technique. It is proposed for the complementary management of residual fragments after fURS or SWL or even asymptomatic stones of less than 5 mm.

Pace et al. [6] showed, in a paper published in 2001, that after SWL, the mechanical percussion and inversion group had a substantially higher stone-free rate than the observation group (40% versus 3%, respectively, *p* < 0.001).

It is therefore proven in these studies that post-urotherapy has a positive effect on the residual fragment rate after SWL or fURS for kidney stone destruction.

Forced diuresis with or without furosemide injection has been performed only in the context of SWL management. No study has ever associated forced diuresis with fURS.

In the article from Chiong et al. [7], published in 2004, evaluating the stone-free rate after furosemide injection post SWL, the radiologically documented complete stone clearance rate at 3 months for the control group was 35.4% and for the experimental group was 62.5% (*p* = 0.006).

A study published in 2015 by A. Ahmed’s team [8] showed a significantly (*p* = 0.030) higher stone-free rate in the furosemide injection group (78.3%) compared to the control group (61.1%) for stones located in the lower calyx after SWL. There were no significant differences between the furosemide injection group and control group in terms of adverse events which were all mild complications.

In the 2017 study by S. Sabharwal et al. [9], evaluating the impact of forced diuresis by injection of 40 mg of furosemide on the stone-free rate, better clearance was found in the experimental group (77.1%) compared to the control group (70.8%) after SWL, despite non-significant results (*p* = 0.49).

In the works of S Sohu et al. [10], published in 2019, after 2 months, the stone-free rate after SWL was much higher in the group with furosemide 40 mg injection and intravenous hyperhydration (77.0% vs 65.3% [*p* < 0.001]). Furthermore, for patients aged ≤ 40 years, the stone-free rate was significantly higher in experimental group than control group, at 89.2% vs 71.4% (*p* < 0.001). There was no significance difference in the complication rates between the furosemide group and the control one.

The study carried out by Kocaaslan et al. [11] investigated the efficacy of forced diuresis with 40 mg furosemide and IV hyperhydration prior to SWL for stones between 6 and 20 mm. No statistically significant difference was found between the groups; the stone-free rate was 69% in the control group and 71% in the treatment group (*p* = 0.758).

The team of Liying Dong et al. [12] carried out a review of the literature and a meta-analysis on the subject in 2020. Six randomized controlled trials containing 1344 patients were included in this meta-analysis, which compared diuretics with placebo on SWL treatment of urolithiasis. In the analysis, they are founding those diuretics on SWL treatment were more effective for the management of urinary stones. Compared with placebo, patients who received diuretics during SWL treatment had significantly higher successful stone clearance rate (odds ratio; 1.73, 95% confidence interval; 1.35 to 2.21, *p* < 0.001).

This systematic review and meta-analysis indicated that diuretics during SWL were effective in the management of urolithiasis with lower risk of complications.

There is no evidence in the literature for the efficacy of adjuvant treatment with forced diuresis after fURS.

The results of these various studies are summarized in Table 1.

**Table 1:** Summary of the various studies

Study	Type of study	Year of publication	Type of treatment		Number of patients		Follow-up	Results
			Control group	Experimental group	Control group	Experimental group		
Chiong et al. [7]	Randomized, controlled	2005	SWL	SWL + IV HH	49	59	3 m	35.4% vs 62.5%, *p* = 0.006 Better elimination of stones with forced diuresis
Ahmed et al. [8]	Randomized, controlled	2015	SWL	SWL + furosemide (20 mg)	100	100	3 m	61.1% vs 78.3%, *p* = 0.03 Better elimination of stones with forced diuresis
Kocaaslan et al. [11]	Randomized, controlled	2015	SWL	SWL + furosemide (40 mg)	72	69	2 w	69% vs 71%, *p* = 0.758 Not significant
Sabharwal et al. [9]	Randomized, controlled	2017	SWL	SWL + furosemide (40 mg)	48	48	2 w	77.1% vs 70.8%, *p* = 0.49 Not significant
Sohu et al. [10]	Randomized, controlled	2019	SWL + IV HH	SWL + IV hyperhydration + furosemide (40 mg)	357	357	2 m	65.3% vs 77%, *p* < 0.001 Better elimination of stones with forced diuresis

*SWL* Extracorporeal shock wave lithotripsy, *HH* Hyperhydration, *IV* Intravenous, *PO* Per os, *w* Weeks; *m* Months

In view of the positive results of forced diuresis after SWL, we propose to carry out the first randomized, controlled, multicentric, superiority trial with a blinding evaluation analyzing forced diuresis with furosemide injection after fURS for laser destruction of kidney stones.

### Objectives

Primary objective of the study:

- To show that the injection of 40 mg of furosemide in slow intravenous (IV) during 10 min, after the procedure, increases the stone-free rate 3 months after a fURS for destruction of kidney stones with laser

Secondary objectives of the study:

- To show that the rate of early post-operative urinary tract infection (UTI) within the first post-operative month after fURS with laser destruction of kidney stones is lower in patients who received forced diuresis with injection of furosemide
- Evaluation of post-operative pain
- Safety of the use of furosemide in patients treated by fURS for renal stone laser destruction
- Effect of the prescription of an alpha-blocker as usual treatment on stone-free rate, which will be assessed in a subgroup analysis
- Evaluation of agreement between the imaging analysis of the urologist and the specialized radiologist

### Trial design

FIRE Stones is a two-parallel group randomized, controlled, multicentric trial with a blinded assessment.

## Methods: participants, interventions, and outcomes

### Study setting

The study will be conducted in 9 French departments of urology, including 8 university hospitals and a private clinic.

### Eligibility criteria

#### Inclusion criteria

Males or females aged 18 to 80 years, with the need to perform a fURS with destruction of the kidney stones with laser for a stones of less than 3 cm, were included.

#### Exclusion criteria

Patient with contra-indication to furosemide, having furosemide as usual treatment, or requiring an injection of aminoglycoside or vancomycin before or during the procedure will be excluded. Participation in other interventional research with an investigational drug or medical device is also an exclusion criterion.

## Interventions

The experimental group will receive intravenous furosemide 40 mg, after the end of the surgical procedure, when the ureteroscope is removed from the renal cavities; the anesthetist or the anesthetist’s nurse will inject the experimental treatment. Furosemide 40 mg diluted in 50 mL NaCl 0.9% will be administered as a single slow (over 10 min) intravenous injection.

In the event of poor tolerance during the 10 min slow intravenous injection, this may be stopped at the discretion of the anesthetic team.

The bottles of furosemide are dedicated to the study and supplied by the sponsor. The furosemide prescription will be issued via a nominative prescription obtained from EnnovClinical (e-CRF) after randomization. The department will attach the two detachable labels from the ampoules used, send the original to the pharmacy, and keep a copy in the patient file.

After discussions between urologists, anesthetists, nephrologists and the pharmacovigilance department at Tours University Hospital, we decided to propose a dose of 40 mg of furosemide [12]. Furosemide is an old drug whose side effects are well known and controlled. Dosages of 20 or 40 mg are very low doses of furosemide. Some patients may take 500 mg of furosemide a day. Undesirable effects are therefore considerably lower for dosages of 20 and 40 mg. As part of a pragmatic study looking for a significant improvement in stone evacuation and the stone-free rate, we felt that the 40 mg dosage was preferable, as it could possibly demonstrate a difference that was not visible with 20 mg furosemide, without increasing the risk of complications associated with the drug. As this was the first study to assess the impact of forced diuresis on stone-free levels after fURS for laser destruction of kidney stones, we felt it was important to take every opportunity to find a significant difference, if any.

Treatment must not be administrated in rapid IV injection. Indeed, we chose to carry out a slow injection because the fast injection of furosemide can involve an ototoxicity being able to go until the temporary or final surdity. This risk increases with the dosage used.

The control group will receive standard of care corresponding to the performance of a fURS without injection of furosemide or other drugs.

## Outcomes

### Primary outcome

The stone-free rate at 3 months of the fURS for renal stone laser destruction will to be evaluated on the low dose abdomino-pelvic CT scan. A centralized review of the images will be performed by two specialized radiologists, in a blinded and crossed way to allow a homogenization of the results.

It was decided to carry out an abdominopelvic CT scan, which is more radiant than a renal ultrasound scan, as this examination is not operator-dependent and can therefore be reviewed by the referring urologist, as well as by several specialized radiologists, to ensure optimal analysis. It was therefore decided to use “low dose” abdominopelvic scans, guaranteeing minimal irradiation (irradiation comparable to a standard X-ray, i.e., 6 times less than a conventional scan), while maintaining optimal analysis of our endpoint [13].

We have designated as “stone-free” patients for whom no calculi were found on follow-up imaging but also if the residual fragments were less than 4 mm [9, 14], as described in the current literature.

### Secondary outcomes

The post-operative UTI will be assessed within 30 days after surgery on the combination of fever higher than 38.5 °C and/or chills and/or clinical symptoms (supra-pubic pain, dysuria, pollakiuria, urgency, urinary burning, back pain radiating to the genitals, hematuria) and/or positive urine culture with a significant bacteriuria threshold (depends on the germ isolated and the gender of the patient) [15].

The post-operative pain will be assessed on visual numerical (from 0 to 10) pain scale in the recovery room, in the service, and at the discharge. The use of opioids will be reported.

Furosemide adverse events will be assessed.

## Safety

The adverse events (AEs) will be self-declared by the patient through the patient logbook. This will be collected at the 3-month post-operative visit. Patients will be questioned about AEs by the investigators.

A telephone call by the clinical research assistant (CRA) will be made 1 month after surgery to check that furosemide is well tolerated once the patient has returned home and also to check for the presence of a post-operative UTI or any other AEs.

## Participant timeline

**Fig. 1:**
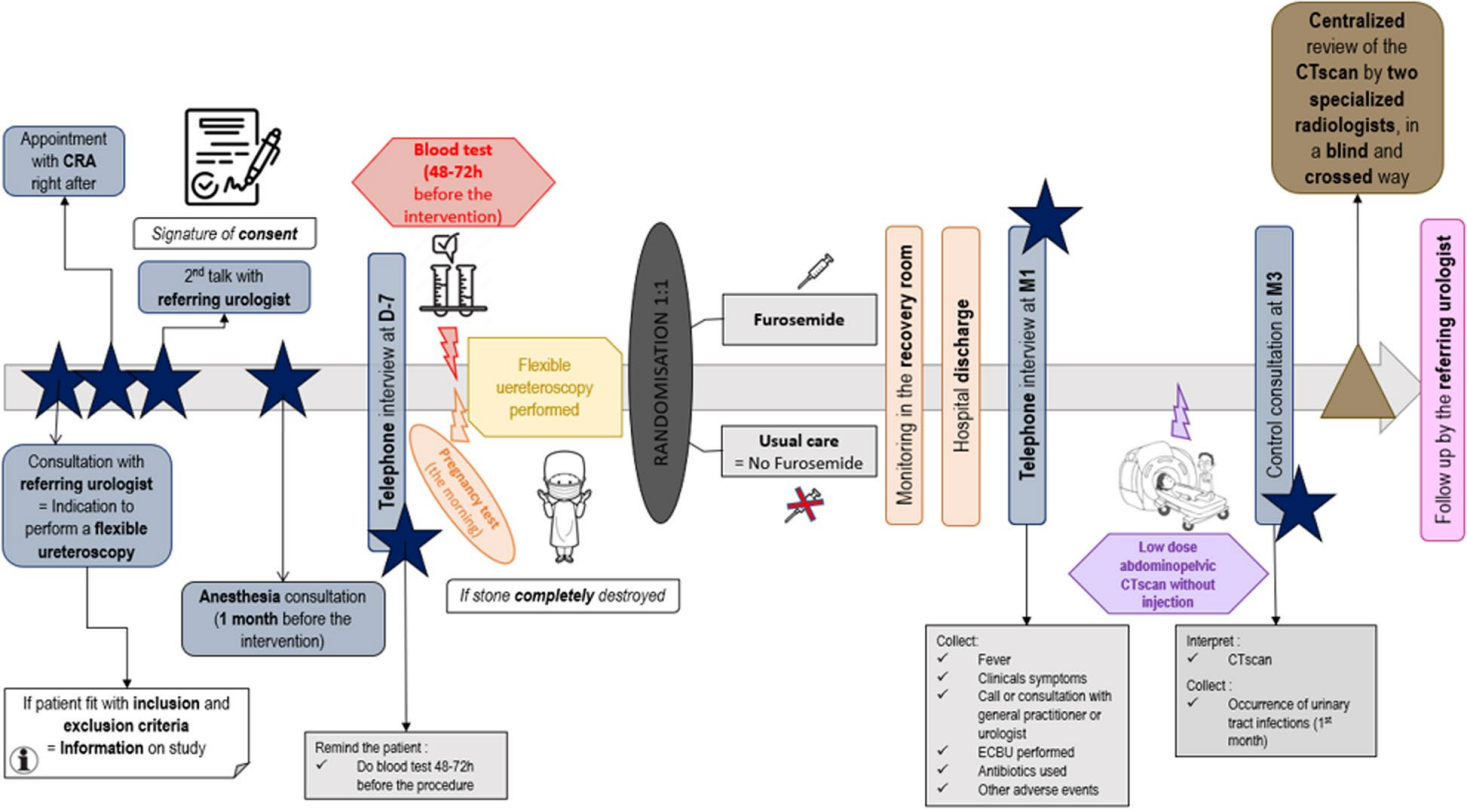
Participant timeline

Duration of participation will be 3 months (± 2 weeks) for each patient. The time schedule of enrolment and visits is in Fig. 1 and Table 2.

**Table 2:** Schedule of enrolment, interventions, and assessments

Timepoint	Inclusion	*− 7 days*	*− 48/72 h*	*D0*	*M1*	*M2*	*M3*
**Before intervention**							
Check inclusion and non-inclusion criteria	X						
Patient information	X						
Signature of consent form	X						
Physical examination	X						
Vital signs with blood pressure monitoring	X						
Phone call		X					
ECBU^a^		X					
Blood test^b^			X				
**Intervention**							
Urinary pregnancy test				X			
Flexible ureteroscopy				X			
Randomization				X			
Furosemide				X			
Clinical examination				X^c^			
Blood pressure				X^c^			
Cardiac frequency				X^c^			
**After intervention**							
Post-operative visit^d^				X			
Follow-up book					X	X	X
Phone call					X	X	
Low dose abdomino-pelvic CT scan							X
**Safety evaluation**							
Adverse events (serious or not)	X	X	X	X	X	X	X

^a^An ECBU may be performed in the event of postoperative urinary tract infection symptoms

^b^Blood test includes a blood count and platelet count, creatinine levels, creatinine clearance, and a blood ionogram. Other parameters may be added at the anesthetist’s request, as is standard practice

^c^Monitoring of 5 h to 7.5 h

^d^Clinical examination, blood pressure, cardiac frequency, evaluation of pain

The patient will be recruited by the urologists on the day when the indication to perform a fURS for the destruction of kidney stones is established. Initial information will be given by the urologist during this consultation. An appointment will be made with a CRA following this consultation to discuss the study in more detail and answer any questions the patient may have, particularly regarding logistical aspects. After this interview with the CRA, the patient will see the urologist again to sign his consent if he agrees to take part in the study, prior to any investigation.

The patient will be called for his/her anesthesia consultation in the month prior to the procedure, as is done in current practice. As it is usually done, an ECBU will be done 1 week before the surgery [14]. A blood ionogram and creatinine will be necessary before inclusion in the study to verify the absence of contra-indications, particularly to furosemide. This blood test should be performed 48–72 h before the surgical procedure in order to be as close as possible to the furosemide injection, if applicable.

The CRA will contact the patient by phone 1 week before the surgery to remind him/her of the necessity to perform the biological check-up 48–72 h before the surgery to verify his/her eligibility for the furosemide injection if necessary.

A urinary pregnancy test will be performed for women of childbearing age on the morning of the procedure.

The randomization will be done at the end of the fURS, if the destruction of the kidney stone is macroscopically complete. The anesthesia team injects furosemide or not, depending on the randomization of the patient.

At post-operative visit, careful monitoring will be carried out in the recovery room by the anesthetic nurses for about 2 h after the operation.

The half-life of furosemide is 1 h to 1.5 h after injection. Considering that the product is completely eliminated from the body after 5 half-lives, a monitoring of 5 h to 7.5 h will be required to ensure optimal monitoring of patients after their injection, which corresponds to the postoperative monitoring in routine practice (in conventional or ambulatory units).

Evaluation of pain will also be assessed by nurses during hospitalization. Pain will be assessed using a numerical pain scale ranging from 0 to 10. This will be done at three time points: in the recovery room, in the service, and at the discharge.

The patient will be followed up for 3 months (± 2 weeks), after the surgery. Patients will use a follow-up book to carry out a self-assessment in particular on the UTI. As usually, patients should consult their general practitioner in the event of sign of infection. However, only the combination of some of these symptoms will be synonymous with a UTI, requiring the expertise of a general practitioner or urologist to distinguish between a real UTI and just the postoperative symptoms caused by the procedure performed in the excretory tract, corresponding to the healing of the urinary mucosa. The ECBU should only be carried out at the request of a doctor (general practitioner or urologist).

A phone call will be performed 1 month after the procedure. We will collect the potential event: fever > 38.5 °C, chills, clinicals symptoms, call or consultation with general practitioner or urologist within 30 days of surgery, ECBU performed within 30 days of surgery, antibiotics within 30 days of surgery. All adverse events presented by the patient since the intervention will be collected.

The patient will have to perform his/her low dose abdomino-pelvic CT scan without injection at 3 months after the operation (± 2 weeks).

We scheduled another phone call by the CRA at about 2 months post-operatively to remind the patient to have his non-injected low-dose abdominal-pelvic CT scan at 3 months (± 2 weeks).

The results of the CT scan should be brought back at the 3-month follow-up consultation with his/her referring urologist. The CT scan data will be interpreted by the urologist and retrieved for an anonymous reading by 2 radiologists in a blind process.

We will also collect the occurrence of UTI in the first post-operation month or other complications, using the patient’s memory and the participant diary noting information.

The CT scans will be collected by the CRA and will be recorded on a dedicated and anonymized platform in order to allow blind evaluation of the images by the 2 specialist radiologists.

A metabolic work-up (blood test, urine test based on a morning sample and 24-h urine collection) is essential for all patients with lithiasis. If the work-up has not already been carried out in the patient’s history of lithiasis, it should be performed postoperatively [14].

At the end of the study, the patient will continue the usual follow-up with his/her referring urologist.

The overview of the study is summarized in Fig. 1 and in Table 2.

## Sample size

The proportion of stone-free patients at 3 months is expected to be 95% in the furosemide group, as compared to 85% in the control group. Indeed, in the literature, the stone-free rate is estimated at 85% [16]. We hypothesize that our treatment will increase this percentage by 10% to have an impact in current. Considering a two-sided type I error level of 5% and a power of 90%, we need to randomize 374 patients.

Because patients will be pre-included, and randomized only at the end of the surgical procedure, we plan to include 416 patients, thus considering that 10 % of them will not be randomized.

## Recruitment

The patient will be recruited by the urologists the day where the indication to perform a fURS for kidney stone destruction is established.

## Methods: assignment of interventions

### Allocation

#### Sequence generation and allocation concealment mechanism

We will use a computer-generated randomization schedule performed by an independent statistician not otherwise involved in patient recruitment or follow-up. Randomization will be stratified by use of double J stent (yes/no), localization of the stone (inferior calix/other), and complete dusting (yes/no). We will use permuted blocks, and block sizes will not be disclosed to the study investigators to ensure concealment.

Randomization procedure will be centralized via a web-based interactive response system (CSonline) managed by the biometrical unit of the Tours University Hospital, thus ensuring allocation concealment.

#### Implementation

Randomization will be performed in the operating room, if the destruction of the stone has been macroscopically complete. Participants will be randomly assigned to experimental group (injection of furosemide) or to control group with a 1:1 ratio allocation. The allocating sequence will be implemented by means of an electronic case report form (e-CRF): once a patient is included.

### Blinding

We plan a pragmatic trial, and we deliberately decided not to use a placebo, to make it much easier to conduct. There is no risk of bias, neither for the performance bias (since the intervention consists in only one injection) nor for the detection bias (since the primary outcome will be centrally assessed, by blinded experts). Therefore, we consider a placebo as useless.

In the end, in the present trial:

- Patients will be blinded: they will not be informed of their allocation at the end of the surgery
- Urologists and anesthesiologists will not be blinded. Indeed, depending of the organization, randomization will be performed either by the urologist or the anesthesiologist or by a nurse. We considered that modifying this organization may be a source of burden, without real advantage. So, the care provider (urologist) is not expected to be blinded
- Radiologists who assess CT scans (i.e., outcome assessor) will be blinded

## Methods: data collection, management, and analysis

### Data collection methods

Table 2 shows data collection according to inclusion and follow-up visits.

### Data management

An e-CRF will be developed by using the Ennov Clinical© software. The e-CRF will be managed in agreement with INSERM CIC 1415 standardized operating procedures (SOP). Data from investigating centers will be entered by using a secure web site monitored by CRA, and queries will be edited by data managers, in agreement with an a priori-specified data-management plan. Blinded review will be performed before locking the database. The database will be locked in agreement with INSERM CIC 1415 SOPs, and data will be extracted in a SAS format or other, according to statistical requirements. Raw data will be stored in XML format.

### Statistical methods

The intention-to-treat (ITT) principle will be applied. Nevertheless, participants who withdraw their consent to study participation will be discarded in case they explicitly refuse that their collected data be used, as required by the French legislation. The number of participants with missing data for each variable of interest will be indicated. Missing data will be managed using a multiple imputation approach. Variables associated to the missingness mechanism will be identified form the study data.

#### Statistical analysis of the primary outcome

Proportion of stone-free patients at 3 months will be compared between the experimental and the control groups using a logistic regression in which stratification variables will be included. The intervention effect will then be expressed as an odds ratio. In agreement with the CONSORT guidelines, the inter-group difference and its 95% confidence interval will also be estimated. For that, a linear model using an identity link function will be estimated. Missing data will be managed using a multiple imputation approach.

#### Statistical analysis of secondary outcomes

Proportion of patients with post-operative UTI within 30 days after surgery will be compared between randomization groups using the same approach as for the analysis of the primary outcome.

Visual numerical scale of pain in the recovery room, in the service, and at discharge will be analyzed using a mixed model. Proportion of patients who received opioids will be compared between randomization groups using the same approach as the one use for the analysis of the primary outcome.

Analysis of the occurrence of furosemide-related events will be descriptive.

#### Ancillary analysis

An evaluation of agreement between the imaging analysis of the urologist and the specialized radiologist will be done considering the primary outcome. Urologists will assess CT scan while the study is going on, while specialized radiologists will assess the CT scan at the end of the study.

We will assess agreement between the imaging analysis of the urologist in center and the centralized reading of the CT scans using the Kappa coefficient and its 95% confidence interval. We will also estimate the agreement rate and its 95% confidence interval and we will study the discordant cases.

#### Sub-group analyses

The effect of the prescription of an alpha-blocker will be looked at considering the primary outcome.

Sub-group analyses will be performed using a modeling approach, by introducing the variable associated to the sub-groups and its interaction with the group variable. A forest plot will be drawn to report the results of these subgroup analyzes.

No interim analysis is planned as part of our study.

## Methods: monitoring

### Data monitoring

A CRA will be responsible for coordination of the study: he/she will be responsible for the logistics of and monitoring the study; producing reports concerning its state of progress, verifying that the e-CRFs are updated (request for additional information, corrections, etc.), importing CT scans and making them anonymous, and transmitting severe AEs to the sponsor. The technician will follow the SOPs.

In view of the low risk presented by the single administration of 40 mg furosemide, no data safety monitoring board (DSMB) is foreseen in this trial. In the same way, no ancillary or post-trial care or compensation is planned for this trial.

### Harms

All AEs will be monitored until they are completely resolved. The investigator will immediately notify the sponsor of any serious AE. The sponsor will report all suspected unexpected serious adverse reaction (SUSARs) to the Eudravigilance (European pharmacovigilance database), French health authorities (ANSM), and the investigators within the regulatory time periods for reporting.

### Auditing

An audit may be performed at any time by sponsor-appointed people who are independent of those responsible for the study. The aim of an audit is to ensure good quality of the study, the validity of results, and that the law and regulations in force are well observed. The investigators agree to comply with the requirements of the sponsor and the relevant authority for an audit or inspection of the study. The audit can apply at all stages of the study, from development of the protocol to publication of results.

## Ethics and dissemination

### Research ethic approval

The sponsor and the investigators undertake to conduct this study in compliance with French law no. 2004–806 of August 9, 2004, and following Good Clinical Practice and the Helsinki Declaration (Ethical Principles for Medical Research involving Human Subjects, Tokyo 2004). The study will be conducted in accordance with this protocol. With the exclusion of emergency situations requiring specific therapeutic actions, the investigators will observe the protocol in all respects, particularly in obtaining consent and the notification and follow-up of serious AEs. The protocol was approved by the institutional review board of the University Hospital Centre of Tours and received authorization from ANSM.

### Protocol amendments

Important protocol modifications will be submitted as well for approval to the Institutional review board of the University Hospital of Tours and will be communicated to coinvestigators.

### Consent and assent

The patient will give their informed signed consent after they have been orally informed of the study and have received a written information form (Additional file 1). These potential participants are informed that they are free to withdraw from the study at any moment.

### Confidentiality

During this research study or when it is over, the information collected on the people taking part in it and forwarded to the sponsor by the investigators (or any other specialized staff member involved) will be made anonymous. Under no circumstances will the uncoded names or addresses of the people concerned appear in any data.

### Access to data

The sponsor is responsible for obtaining agreement from all the parties involved in the study in order to guarantee direct access, in all the sites where the study is being conducted, to source data, source documents, and reports, to control their quality, and to audit them. The investigators will make available to people with a right of access to these documents, according to the legislative and regulatory provisions in force (articles L.1121–3 and R.5121–13 of the French Public Health Act), the documents and individual data strictly necessary for monitoring, carrying out quality control and auditing the biomedical research.

### Dissemination policy

INSERM CIC 1415 Tours will analyze the data provided by the study centers. Results will be displayed in a written report that will be submitted to the sponsor. At the end of the analysis, results will be published in ClinicalTrials.gov. The international rules for writing and publication (Vancouver Agreement, February 2006) will be followed. In accordance with law no. 2002–303 of March 4, 2002, patients will be informed, at their request, about the overall results of the study [17].

### SPIRIT

This protocol has been written in accordance with the Standard Protocol Items: Recommendations for Interventional Trials (SPIRIT) guidelines. The SPIRIT checklist is in Additional file 2. The SPIRIT figure is in Table 2.

## Discussion

Lithiasis is a public health problem. It affects about 10% of the general population. This prevalence is increasing (multiplied by 3 in 40 years) [1], partly due to changes in the population’s eating habits over the years. The lithiasis patient is a patient with a chronic disease requiring annual follow-up and who may suffer from multiple recurrences, with a recurrence rate at 5 years of 50% [1, 2].

Recurrences are partly due to residual fragments left in the kidneys at the end of the operation.

As far as residual fragments are concerned, their presence leads to an early recurrence of stones because they form the bed for a new aggregation of crystals in the kidneys. Being able to reduce the rate of residual fragments in patients with the use of furosemide at the end of the intervention therefore seems essential in the management of recurrences in our patients. This will also improve our patients’ quality of life. Indeed, lithiasis disease leads to chronic pain associated with acute pain that motivates consultations to the emergency for specialized management.

This FIRE Stones project will be the first to study the impact of furosemide injection after fURS for renal stones.

fURS for stones are performed in common practice by most urologists, both independent and practicing in hospital. In fact, more than 40,000 fURS for stones are performed per year in France. It means that the number of patients will be sufficient to conduct the study. The selected centers of this study are used to recruit in clinical studies and frequently perform this procedure. Depending on the center, an average of 90 to 200 fURS for kidney stone destruction are performed per year. Moreover, this is a pragmatic study that fits perfectly into the current practice of urologists. Its realization does not compromise the organization of the services that will participate, thus allowing a better adhesion to the protocol.

## Trial status

The current version of the protocol is V1.2, dated 4 September 2023. The protocol is currently recruiting in 9 French centers. This study has been open since the end of November 2023, with centers opening gradually. Currently, 3 centers are open. No patients have yet been included. The recruitment is anticipated to end in December 2025, with follow-up completed in March 2026.

### Supplementary Information

Additional file 1. Informed consent materials.

Additional file 2. SPIRIT checklist.

## Data Availability

The datasets generated during the current study are available from the corresponding author on reasonable request.

## Abbreviations

AEs: Adverse events
AFU: Association Française d’Urologie
ANSM: Agence Nationale de Sécurité du Médicament et des produits de santé
CRA: Clinical research assistant
DSMB: Data safety monitoring board
e-CRF: Electronic case report form
fURS: Flexible ureteroscopy
ITT: Intention-to-treat
IV: Intravenous
PCNL: Percutaneous nephrolithotomy
SOP: Standardized operating procedures
SUSAR: Suspected Unexpected Serious Adverse Reaction
UTI: Urinary tract infection
SWL: Extra-corporeal shock wave lithotripsy
