# Supplementary material for: FIRE Stones: impact of forced diuresis on the residual fragment rate after flexible ureteroscopy for destruction of kidney stones with laser—protocol for a randomized controlled two-parallel group multicenter trial with blinding evaluation
Source: Trials. 2024 Jul 4;25:455. doi: 10.1186/s13063-024-08309-0 (PMC11225219; doi:10.1186/s13063-024-08309-0)
Supplement: Supplementary file 1 — Additional file 1. Informed consent materials. [file 13063_2024_8309_MOESM1_ESM.pdf]

DOCUMENT D'INFORMATION DE LA RECHERCHE

Version 1.1 du 10/08/2023

Etude FIRE Stones :

Impact de la diurèse forcée sur le taux de sans fragment résiduel après urétéroscopie souple pour destruction de calculs rénaux par laser : essai randomisé contrôlé en deux groupes parallèles multicentrique avec évaluation en aveugle

Promoteur de la recherche :

CHRU de Tours - 2, boulevard Tonnellé 37044 Tours Cedex 9

Investigateur coordonnateur :

Dr. Marie-Lou LETOUCHE
Téléphone : +33 2 34 38 95 63
Service d'urologie
Hôpital Bretonneau – CHRU de Tours
2 bd Tonnellé, 37044 Tours Cedex 9

Madame, Monsieur,

Vous avez été invité(e) à participer à la recherche interventionnelle intitulée FIRE Stones organisée par le CHRU de Tours. Ce document a pour but de vous fournir les informations écrites nécessaires à votre décision. Il est important que vous compreniez le déroulement de cette étude, ses objectifs et ce qu'elle impliquera pour vous en termes de bénéfices, risques et gênes potentiels. Lisez-le attentivement et n'hésitez pas à poser toutes les questions qui vous sembleront utiles à sa bonne compréhension. Vous pouvez prendre le temps nécessaire pour décider si vous souhaitez participer à cette recherche.
Sachez également, qu'en application des dispositions de l'article L1111-6 du code de la santé publique, vous avez la possibilité de vous faire assister par une personne de confiance afin de vous aider dans votre décision.
Si vous acceptez de participer, vous devrez compléter et signer le formulaire de consentement.

QUEL EST L'OBJECTIF PRINCIPAL DE CETTE ÉTUDE ?

Vous êtes invité(e) à participer à l'étude FIRE Stones car votre urologue a retenu l'indication de réaliser une urétéroscopie souple pour le traitement de vos calculs rénaux. Cette intervention permet la destruction des calculs en fragments qui peuvent ensuite être évacués par les voies urinaires. Des micro-fragments et poussières créés lors de cette destruction peuvent subsister dans le rein et créer un nid pour une nouvelle formation de calculs. Des traitements ont été décrits pour augmenter l'expulsion des fragments résiduels, néanmoins leur impact après une urétéroscopie souple reste peu clair et principalement théorique.

Le Furosémide est un diurétique de l'anse, connu pour augmenter significativement le débit urinaire, améliorant donc les chances d'évacuer les fragments résiduels du calcul détruit lors de l'urétéroscopie souple. Nous proposons donc de réaliser une étude dont l'objectif principal est de montrer que la diurèse forcée par injection de 40 mg de Furosémide après une urétéroscopie souple pour destruction de calculs rénaux augmente le taux de sans fragment résiduel.

QUE SE PASSERA-T-IL EN PARTICIPANT A LA RECHERCHE ?

Si vous présentez les critères requis et acceptez de participer à l'étude, vous signerez un formulaire de consentement avant toute intervention dans le cadre de l'étude. La durée de votre participation est de 3 mois.

- 7 jours avant l'urétéroscopie souple, <u>vous serez contacté(e) par téléphone</u> pour vous rappeler qu'un bilan biologique est à réaliser 48-72h avant l'intervention.
- Suite à l'intervention, si celle-ci a permis une destruction complète des calculs, <u>un tirage au sort sera réalisé</u> pour savoir si vous serez affecté(e) au groupe expérimental qui recevra 40 mg de Furosémide ou au groupe contrôle qui ne recevra pas de Furosémide. L'injection intra-veineuse de Furosémide sera réalisée après l'intervention.

- 1 mois après l'urétéroscopie souple, vous serez contacté(e) par téléphone afin de recueillir la survenue d'éventuel(s) événement(s) indésirable(s)
- 3 mois après l'intervention, vous devrez réaliser un scanner et un bilan biologique dans un cabinet de ville et transmettre les images et résultats au médecin investigateur lors de votre consultation dans le service d'urologie. Vous recevrez un appel 1 mois avant, vous rappelant la nécessité de réaliser ces examens.

QUELS SONT LES RISQUES ET CONTRAINTES LIÉS À LA PARTICIPATION ?

Le médicament utilisé dans cette étude, le Furosémide, est un médicament connu depuis de nombreuses années. Ces effets indésirables sont donc bien connus et maitrisés. De plus, la faible posologie choisie dans notre étude (40 mg) et le fait qu'il s'agisse d'une administration unique diminuent le risque de survenue d'effets indésirables.

Toutefois, le furosémide peut avoir un effet négatif sur la douleur post-opératoire. En effet, son ajout va permettre une meilleure élimination des micro-fragments qui peuvent se bloquer dans l'uretère. Ceci peut provoquer des douleurs de coliques néphrétiques.

Aussi, il a été choisi de réaliser un scanner abdominopelvien, plus irradiant qu'une échographie rénale, car cet examen n'est pas opérateur dépendant, permettant donc une relecture par l'urologue référent, mais aussi par plusieurs radiologues spécialisées afin de garantir une analyse optimale. Il a donc été décidé de réaliser des scanners abdominopelviens « low dose » garantissant une irradiation minime (irradiation comparable à une radiographie standard, soit 6 fois moins qu'un scanner usuel), mais en conservant une analyse optimale de notre critère de jugement.

QUELS SONT LES BÉNÉFICES DE LA PARTICIPATION À L'ÉTUDE ?

La participation à cette étude ne présente aucune garantie de bénéfice personnel. En participant, vous nous aiderez à déterminer l'intérêt du Furosémide dans l'augmentation du taux de sans fragment résiduel lors d'une urétéroscopie souple pour destruction de calculs rénaux par laser. En effet, il n'y a pas de recommandations thérapeutiques claires à ce sujet alors qu'il s'agit d'un problème de santé publique, touchant environ 10% de la population générale. Cette maladie chronique nécessite un suivi annuel et un taux de récidive à 5 ans de 50%. Elle entraîne des douleurs chroniques associées à des douleurs aiguës.

L'utilisation de ce médicament ne changera pas la réalisation du geste chirurgical qui aura les mêmes modalités dans les deux groupes d'étude. En effet, le tirage au sort pour savoir si vous faites partie du groupe avec ou sans injection de Furosémide ne se fera qu'à la fin de l'intervention, et seulement si les calculs sont macroscopiquement complétement détruits. Le cas échéant, l'injection de Furosémide sera faite après que l'intégralité de l'intervention ait été réalisée.

Pouvoir augmenter le taux de sans fragment résiduel chez les patients avec l'utilisation de Furosémide en fin d'intervention pourrait réduire le risque de récidive et donc améliorer la qualité de vie de nombreux patients.

LA PARTICIPATION A L'ETUDE COUTE-T-ELLE QUELQUE CHOSE ?

Votre participation à cette étude n'engendrera aucun frais supplémentaire pour vous.
Les frais de transport (transport en commun ou véhicule personnel) nécessaires à la réalisation de la recherche, pour le bilan biologique à réaliser avant l'intervention et le scanner à 3 mois post-opératoire, vous seront remboursés sous remise de justificatifs à envoyer à la DRCI du CHRU de Tours. A noter que pour pouvoir participer à cette étude, vous devez être affilié(e) ou bénéficier d'un régime de sécurité sociale.

EST-CE QU'IL EST POSSIBLE DE RENONCER A SA PARTICIPATION ?

Votre participation est entièrement volontaire. Vous êtes donc libre d'accepter ou de refuser de participer sans que votre décision n'ait de conséquence sur vos soins médicaux. Si vous acceptez, vous pourrez à tout moment renoncer à votre participation, sans justification, et sans que cela ne porte préjudice à la suite de votre prise en charge ou à vos relations avec votre médecin. A la fin de votre participation à l'étude, y compris si celle-ci est interrompue, vous serez traité(e) selon les recommandations en vigueur. En accord avec l'article L1122-1-1 du code de la santé publique, les données obtenues jusqu'à votre retrait de consentement seront exploitées au moment des analyses sauf demande expresse de votre part.

TRAITEMENT DES DONNÉES ET DROIT D'ACCES

Dans le cadre de cette recherche, un traitement informatisé de vos données de santé va être mis en œuvre sous la responsabilité du CHRU de TOURS, en tant que Promoteur de la recherche, et dans le respect de la réglementation en vigueur (*Règlement européen (UE) 2016/679 du 27 avril 2016 relatif à la protection des données personnelles (RGPD) et la loi n° 78-17 du 6 janvier 1978 relative à l'informatique, aux fichiers et aux libertés modifiée).* Ce traitement, conforme à la méthodologie de référence MR001 de la CNIL selon l'engagement du Promoteur, permettra d'analyser les résultats au regard de l'objectif qui vous a été présenté. Il est donc nécessaire aux fins de cette recherche scientifique (art.9 RGPD) et aux fins des intérêts légitimes poursuivis par le Promoteur (art.6 RGPD).

Vos données seront identifiées par un code (numéro de centre suivi du numéro d'inclusion dans le centre) sans mention de vos noms et prénoms. Elles pourront dans des conditions assurant leur confidentialité, être transmises au Promoteur ou aux personnes agissant pour son compte. Avec votre accord, vos données codées pourront être réutilisées pour d'autres recherches portant sur la même thématique. De nouvelles recherches pourront ainsi être menées par le promoteur, seul, ou avec un ou plusieurs établissements publics ou privés (y compris hors Union-Européenne), ou uniquement par ces derniers. Vous disposez des mêmes droits pour ces recherches et pourrez donc vous opposer à une telle utilisation. Vous disposez à tout moment d'un droit d'accès, de rectification, d'effacement, de limitation ou d'opposition au traitement des données. Ces droits peuvent s'exercer auprès des investigateurs de cette recherche ou du Promoteur, par l'intermédiaire de son délégué à la protection des données (dpo@chu-tours.fr). Vous pouvez également introduire une réclamation auprès d'une autorité de contrôle et de protection des données personnelles (CNIL pour la France ; 01.53.73.22.22 / CNIL - 3 Place de Fontenoy - TSA 80715 - 75334 Paris Cedex 07 / https://www.cnil.fr/fr/webform/adresser-une-plainte). Le promoteur, par l'intermédiaire de son délégué à la protection des données s'assurera de la conformité du traitement des données conformément aux dispositions du Règlement (UE) 2016/679. Les données collectées seront conservées pour une durée conforme à la réglementation en vigueur (25 ans).

QUI A APPROUVÉ LA RECHERCHE ?

En application des dispositions de l'article L1121-4 du code de la santé publique, les modalités de cette recherche ont été soumises à un Comité de Protection des Personnes (CPP) et à l'Agence Nationale de Sécurité du Médicaments et de produits de santé (ANSM). Le CPP, qui a notamment pour mission de vérifier les conditions requises pour la protection et le respect de vos droits, a donné un avis favorable et l'ANSM a donné son autorisation après évaluation des bénéfices et des risques attendus afin de garantir votre sécurité. En application des dispositions de l'article L1121-10 du code de la santé publique, le Promoteur de cette recherche, le CHRU de Tours, a contracté une assurance auprès de la compagnie Relyens Mutual Insurance localisée au 18 rue Edouard Rochet – 69372 Lyon Cedex 08 et joignable au 04 72 75 50 25. A noter que les personnes ayant subi un préjudice après participation à une recherche peuvent faire valoir leurs droits auprès des commissions régionales de conciliation et d'indemnisation des accidents médicaux (article L1142-3 du code de la santé publique). Si vous estimez avoir subi un préjudice, vous devrez donc contacter le médecin qui vous a proposé cette étude.

EN CAS DE QUESTIONS :

Le médecin qui vous a proposé cette recherche est à votre disposition pour vous fournir toutes informations complémentaires. Si vous le souhaitez, vous pouvez contacter directement le coordonnateur de cette recherche : le Dr. Marie-Lou LETOUCHE dont vous trouverez les coordonnées en première page de ce document.
En application des dispositions de l'article L1122-1 du code de la santé publique, les résultats globaux de l'étude pourront vous être communiqués en fin d'étude à condition que vous en fassiez la demande auprès du coordonnateur.

Egalement, le résumé des résultats sera disponible sur une base de données européenne, dans un délai de 12 mois après la fin de l'étude, via l'adresse internet ci-dessous. https://euclinicaltrials.eu/search-for-clinical-trials

CHRU HÔPITAUX DE TOURS

FORMULAIRE DE CONSENTEMENT
Version 1.1 du 10/08/2023

Etude FIRE Stones :

Impact de la diurèse forcée sur le taux de sans fragment résiduel après urétéroscopie souple pour destruction de calculs rénaux par laser : essai randomisé contrôlé en deux groupes parallèles multicentrique avec évaluation en aveugle

Je déclare avoir pris connaissance des informations orales et écrites qui m'ont été données sur l'objectif et le déroulement de cette recherche ainsi que les risques potentiels et les contraintes liées à ma participation.

J'ai eu la possibilité de poser toutes les questions que je souhaitais et de recevoir les réponses adaptées de l'investigateur. J'ai également compris que je pouvais refuser de participer sans que cela ne porte préjudice à la suite de ma prise en charge. Ce consentement ne décharge pas l'investigateur et le Promoteur de leurs responsabilités à mon égard, je conserve donc tous les droits garantis par la loi. Et je peux à tout moment retirer mon consentement sans justification et sans conséquence sur la qualité des soins que je continuerai à recevoir.

J'ai noté que mes données de santé recueillies lors de cette recherche demeureront strictement confidentielles et pourront être consultées par l'investigateur, ses collaborateurs, des personnes mandatées par le Promoteur ou les Autorités de Santé, astreintes au secret professionnel.

J'accepte le traitement informatisé de mes données en conformité avec la réglementation en vigueur. J'ai pris connaissance de mes droits d'accès, de rectification, d'effacement, de limitation ou d'opposition à ce traitement.

J'ai compris que je pourrai à tout moment demander des informations complémentaires à l'investigateur qui m'a proposé de participer à cette recherche. Et si j'en fais la demande, les résultats globaux de la recherche pourront m'être communiqués en fin d'étude.

Compte tenu des informations qui m'ont été délivrées et du temps de réflexion pour prendre ma décision, j'accepte librement et volontairement de participer à cette recherche. J'atteste être affilié(e) ou bénéficier d'un régime de sécurité sociale.

❑ J'accepte OU ❑ Je n'accepte pas que mes données personnelles codées soient utilisées pour d'autres recherches sur la même thématique, exclusivement à des fins scientifiques sachant que je peux à tout moment retirer mon accord

Partie à remplir par la personne se prêtant à la recherche	
Nom, prénom :	***Signature :***
Date :	
Partie à remplir par le médecin investigateur	
Nom, prénom :	***Signature :***
Date :	
